# Preclinical Evaluation of a Lead Specific Chelator (PSC) Conjugated to Radiopeptides for ^203^Pb and ^212^Pb-Based Theranostics

**DOI:** 10.3390/pharmaceutics15020414

**Published:** 2023-01-26

**Authors:** Mengshi Li, Nicholas J. Baumhover, Dijie Liu, Brianna S. Cagle, Frédéric Boschetti, Guillaume Paulin, Dongyoul Lee, Zhiming Dai, Ephraim R. Obot, Brenna M. Marks, Ibrahim Okeil, Edwin A. Sagastume, Moustafa Gabr, F. Christopher Pigge, Frances L. Johnson, Michael K. Schultz

**Affiliations:** 1 Viewpoint Molecular Targeting, Inc., 2500 Crosspark Road, Coralville, IA 52241, USA; 2 CheMatech-MDT, 21000 Dijon, France; 3 Department of Physics and Chemistry, Korea Military Academy, Seoul 01805, Republic of Korea; 4 Department of Chemistry, The University of Iowa, Iowa City, IA 52240, USA; 5 Department of Radiology, Weill Cornell Medicine, New York, NY 10021, USA; 6 Department of Radiology, The University of Iowa, Iowa City, IA 52246, USA; 7 Department of Radiation Oncology, The University of Iowa, Iowa City, IA 52246, USA

**Keywords:** Lead-212, Bismuth-212, Lead-203, chelators, stability, theranostics

## Abstract

^203^Pb and ^212^Pb have emerged as promising theranostic isotopes for image-guided α-particle radionuclide therapy for cancers. Here, we report a cyclen-based Pb specific chelator (PSC) that is conjugated to tyr^3^-octreotide via a PEG_2_ linker (PSC-PEG-T) targeting somatostatin receptor subtype 2 (SSTR2). PSC-PEG-T could be labeled efficiently to purified ^212^Pb at 25 °C and also to ^212^Bi at 80 °C. Efficient radiolabeling of mixed ^212^Pb and ^212^Bi in PSC-PEG-T was also observed at 80 °C. Post radiolabeling, stable Pb(II) and Bi(III) radiometal complexes in saline were observed after incubating [^203^Pb]Pb-PSC-PEG-T for 72 h and [^212^Bi]Bi-PSC-PEG-T for 5 h. Stable [^212^Pb]Pb-PSC-PEG-T and progeny [^212^Bi]Bi-PSC-PEG-T were identified after storage in saline for 24 h. In serum, stable radiometal/radiopeptide were observed after incubating [^203^Pb]Pb-PSC-PEG-T for 55 h and [^212^Pb]Pb-PSC-PEG-T for 24 h. In vivo biodistribution of [^212^Pb]Pb-PSC-PEG-T in tumor-free CD-1 Elite mice and athymic mice bearing AR42J xenografts revealed rapid tumor accumulation, excellent tumor retention and fast renal clearance of both ^212^Pb and ^212^Bi, with no in vivo redistribution of progeny ^212^Bi. Single-photon emission computed tomography (SPECT) imaging of [^203^Pb]Pb-PSC-PEG-T and [^212^Pb]Pb-PSC-PEG-T in mice also demonstrated comparable accumulation in AR42J xenografts and renal clearance, confirming the theranostic potential of the elementally identical ^203^Pb/^212^Pb radionuclide pair.

## 1. Introduction

Peptide-targeted radionuclide therapy (PTRT) for cancer has gained considerable traction in oncology [1,2]. As a class of drug compounds, peptides possess properties that make them particularly well-suited for this application. Often, a selective peptide binding domain for a cell surface antigen can be identified based on a native cognate peptide (e.g., somatostatin analogs used for targeting the somatostatin receptor subtype 2) [3,4], or via high throughput peptide display libraries that identify amino acid sequences that bind cell surface antigens with high affinity and selectivity [5,6,7]. In each of these paradigms of radiopeptide development, the identified peptide binding moiety is modified to include a chelator that is designed to enable radiolabeling with the radionuclide(s) of interest to provide a suitable radiopeptide conjugate that can be used for imaging and therapy. In addition to the chemical composition of radiopeptide conjugates, the choice of radionuclides for imaging and therapy plays a critical role in the design of radiopharmaceuticals. Over the past several years, α-emitters have received considerable attention, owing to potential advantages over β-emitters [8,9,10]. Fundamentally, the advantage of α-emitters lies in the higher linear energy transfer (LET) (100 keV/µm) they display versus β-particles and a concomitant increase in ionizations (primary and secondary) along the short path length of their track in the cellular microenvironment in tissue [11]. The deposition of high LET radiation over a short pathlength generates an increase in the incidence of DNA double-strand breaks (DSB), improved tumor-cell-specific killing, and improved relative biological effectiveness (RBE) [11,12,13].

Of the radionuclides that can be practically produced for α-particle based theranostics, ^212^Pb/^203^Pb is the only available elementally identical radionuclide pair. In this context, ^212^Pb (Table 1; half-life t_1/2_ 10.6 h) is generally produced using a ^224^Ra/^212^Pb generator system that enables nimble, on-demand production of ^212^Pb based radiopharmaceuticals [14,15,16]. The decay half-life of ^212^Pb matches well with the relatively shorter biological half-life of small peptides, compared with the long biological half-lives of antibodies [17]. For the imaging component of the theranostic pair, cyclotron-produced γ-emitting radionuclide ^203^Pb can be used as an elementally identical imaging surrogate for ^212^Pb [18,19]. This property provides confidence that predictions made using ^203^Pb SPECT imaging accurately represent the expected pharmacokinetics of the therapeutic ligand. The nuclear data of ^203^Pb, ^212^Pb and progeny are summarized in Table 1. 

Potential ambiguity in these predictions arises due to the nature of the ^212^Pb decay series. The decay of ^212^Pb to ^212^Bi might lead to a decoupling of the progeny ^212^Bi from the chelator, adding uncertainty to the biodistribution of radioactivity, especially the α-emitters (i.e., ^212^Bi, ^212^Po) [20,21,22,23,24]. Using particle and heavy ion transport code system (PHITS) modeling, we have shown that α-particles contribute to more than 90% of dose deposition in 10 µm diameter single cells and 1 cm diameter micro-metastasis, while the dose from β-particles becomes more significant as tumor size increases [25]. The extremely short half-life of ^212^Po (0.3 µ seconds) largely limits its relocation from parent radionuclides, whereas the significantly longer half-life of ^212^Bi (1 h) allows for potential redistribution once it is released from chelation. Most Pb based radiolabeling has been reported using semicarbazone- and amide-based chelators DOTA (1,4,7,10-tetraazacyclododecane-1,4,7,10-tetraacetic acid) and TCMC (1,4,7,10-Tetrakis(carbamoylmethyl)-1,4,7,10-tetraazacyclododecane) [26,27,28,29,30]. DOTA and TCMC are derived from the aza-crown ether cyclen, with four carboxymethyl groups on DOTA and four acetamide groups that decorate the tetraaza core. It has been reported that the electron conversion occurring with the β decay of ^212^Pb results in a hyperoxidized state of ^212^Bi that potentially breaks the ^212^Bi-chelator bonds, causing release of free ^212^Bi^3+^ from these chelators [22,31]. For example, previous studies reported that approximately 36% of ^212^Bi^3+^ was released from a DOTA chelator [23] and 16% of ^212^Bi^3+^ was ejected from the TCMC chelator in an anti-CD37 radioimmunoconjugate [32]. When labeled with Pb(II), [Pb]DOTA displays a net formal charge of −1 or −2, whereas [Pb]TCMC has a net formal charge of +2 due to the neutral charge of TCMC. In this study, we report a Pb specific chelator (i.e., PSC), consisting of a mixture of carboxy and acetamide ligand groups with the acetamide group on the 7′ position of cyclen (i.e., para to conjugates). This chelator was designed for coupling Pb(II) radiometal with zero net formal charge. With PSC conjugated on a tyr^3^-octreotide (TOC) via polyethylene glycol linker (i.e., PSC-PEG-T), we show that PSC-PEG-T not only enables rapid radiolabeling of ^212^Pb and ^212^Bi, but also preserves both ^212^Pb and ^212^Bi daughter coupling, thereby minimizing the potential for generation of free ^212^Bi^3+^ daughter while in formulation and the redistribution of free ^212^Bi^3+^ in the in vivo setting. 

## 2. Materials and Methods

### 2.1. Materials and Reagents

^224^Ra/^212^Pb generator (VMT-a-GEN) was manufactured by Viewpoint Molecular Targeting, Inc. (Coralville, IA, USA) using ^228^Th stock obtained from the US Department of Energy Oak Ridge National Laboratory (Oak Ridge, TN, USA). ^203^PbCl_2_ was provided by the Cyclotron Facility at the University of Alberta (Edmonton, AB, Canada). ^68^GaCl_3_ was kindly provided by Dr. David Dick from the Department of Radiology at the University of Iowa (Iowa City, IA, USA). H-threoninol(But)-2-Cl-Trt-resin, Fmoc-protected amino acids, Fmoc-NH-PEG_2_-propionic acid and [Bis(dimethylamino)methylene]-1H-1,2,3-triazolo [4,5-b]pyridinium 3-oxide hexafluorophosphate (HATU) were purchased from AnaSpec (Fremont, CA, USA). Instant thin layer chromatography (iTLC) paper was purchased from Agilent (Santa Clara, CA, USA). Pb and RE2 resins were provided by Eichrom Technologies (Lisle, IL, USA). Empty polypropylene SPE tubes with 20 μm PE frits were obtained from Millipore Sigma (Burlington, MA, USA). Sep-Pak^®^ C18 cartridges were purchased from Waters Corporation (Milford, MA, USA). Other chemicals were purchased from Thermo Fisher Scientific (Waltham, MA, USA). CD1-Elite SOPF mice were purchased from Charles River Laboratory (Wilmington, MA, USA). Athymic nude mice were obtained from Envigo (Indianapolis, IN, USA). SSTR2-positive rat pancreatic cancer cell line AR42J was purchased from ATCC (Manassas, VA, USA).

### 2.2. Synthesis of Lead Specific Chelator (PSC) and Chelator-Conjugated Peptides

**Figure 1:**
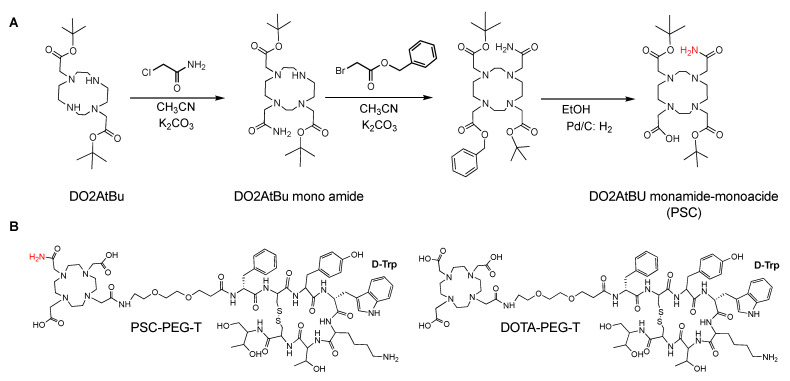
(**A**) Structure and synthesis scheme of PSC chelator; (**B**) Structure of PSC or DOTA conjugated tyr3-octreotide analogue PSC-PEG-T and DOTA-PEG-T.

PSC chelator was synthesized based on DO2AtBu precursor via DO2AtBu mono amide (Figure 1A). First, 50.0 g of DO2AtBu (0.12 mol) was dissolved in 2 L of CH_3_CN. Then, 28.7 g of K_2_CO_3_ (0.21 mol) was added in the solution and the mixture was stirred at room temperature. Next, 9.9 g of 2-chloroacetamide (0.10 mol) was dissolved in 500 mL of CH_3_CN and was added dropwise in the mixture. Solution was stirred at room temperature for 24 h. Mixture was filtered off and solvent evaporated. The residual yellow oil was taken up with diethyl ether and the formation of a precipitate was observed. After filtration, the solid was washed once again with diethyl ether and recrystallized in CH_3_CN to yield DO2AtBu mono amide as a white powder (20.4 g, yield 43%). Filtered DO2AtBu mono amide was characterized by ^1^H NMR (300 MHz, CDCl_3_, 298K). The DO2AtBu mono amide was characterized by ^1^H NMR (300 MHz, CDCl_3_, 298K): d (ppm) 1.44 (s, 18H); 2.66 (s (br), 8H); 2.76 (s (br), 4H); 2.84 (s (br), 4H); 3.06 (s, 2H); 3.28 (s, 4H); 5.33 (s, 1H); 8.29 (s, 1H). Calculated elemental composition for C_22_H_43_N_5_O_5_, 0.6H_2_O was C: 56.41%; H: 9.51%; N: 14.95%; The actual elemental analysis found C: 56.47%; H: 9.71%; N: 14.85%.

DO2AtBu mono amide (0.02 mole; 10.0 g) was dissolved in 100 mL of CH_3_CN. Then, 9.1 g of K_2_CO_3_ (0.07 mole) was added in the solution and the mixture was stirred at room temperature. Next, 5.0 g of benzyl bromoacetate was added to mixture and the solution was stirred at room temperature for 12 h. The mixture was filtered off and solvent evaporated to obtain a pale-yellow oil which was dissolved in 100 mL of ethanol with 10 mL of water. Hydrogenation was conducted by adding 0.1 g Pd/C (palladium on carbon) and mixing under hydrogen atmosphere. After 12 h, Pd/C was removed by filtration and solvents evaporated. Residue was taken up with acetone. The obtained precipitate was purified by filtering off, and PSC was obtained as a colorless solid (5.6 g, yield 50%). Purity (98.5%) and identity of final PSC product were characterized by LC-MS on Thermo Scientific U3000 equipped with a DAD for UV spectrometer and MSQ Plus mass spectrometer, running 5% H_2_O with 0.1% TFA over acetonitrile over 1.5 min on Kinetex C-18 column 2.6 µm, 100A, 50 × 2.1 mm (Phenomenex, Torrance, CA, USA) at 0.5 mL min^−1^. ^1^H and ^13^C NMR characterization of PSC was conducted. ^1^H NMR (600 MHz, DMSO, 323 K): d (ppm) 1.42 (s, 18H); 2.56 (s (br), 4H); 2.75 (s (br), 4H); 2.93 (s (br), 4H); 3.05 (s, 2 H); 3.16 (s (br), 4H); 3.35 (s, 4H); 3.40 (s, 2H) 6.80 (s, 1H); 7.59 (s, 1H).^13^C NMR (150 MHz, DMSO, 323 K): d (ppm) 27.7; 47.6; 49.7; 52.1; 52.2; 54.6; 56.6; 57.5; 80.3; 166.4 (br); 170.0; 172.5. Calculated elemental composition of PSC (C_24_H_45_N_5_O_7_) was 2.7 H_2_O C: 51.08%; H: 9.00%; N: 12.41%; the actual elemental analysis demonstrated C: 50.85%; H: 9.39%; N: 12.16%.

PSC or DOTA was conjugated to the N-terminus of octreotide analogue TOC via PEG2 linker (Figure 1B). PSC-PEG-T was prepared by standard Fmoc procedures on a H-threoninol (But)-2-Cl-Trt-resin employing multiple coupling cycles for all amino acids. Following the coupling of the N-terminal amino acid (Fmoc-Nle-OH) on the linear peptide, Fmoc-PEG_2_-propionic acid was coupled to the N-terminus and deprotected prior to chelator coupling. Conjugation of DOTA or PSC to PEG-T was conducted by adding five equivalents of protected DOTA or PSC to sidechain-protected peptides on resin utilizing HATU/HOBt during the coupling reaction. Completed reaction was monitored by Kaiser test. Upon completion of the reaction, the resin was washed with DMF, DCM and methanol. PSC-PEG-T and DOTA-PEG-T peptides were cyclized via I_2_ oxidation by treating peptides with 20 equivalents of I_2_ in DMF. All peptides were removed from resin and side chain residues were deprotected by incubation in TFA/H_2_O/TIS (95/2.5/2.5%) cleavage cocktail for 2 h, followed by precipitation in ice cold diethyl-ether. The precipitate was centrifuged at 3000 rpm for 10 min at 4°C and the supernatant was decanted. The crude peptide pellet was reconstituted in ultrapure water and lyophilized, followed by purification into single species on semipreparative RP-HPLC. Purified peptides were reconstituted in water and quantified by measuring the absorbance at 280 nm. The purity of all peptides was >95% as analyzed by RP-HPLC (Agilent 1260). Final characterization of peptides was performed by LC-MS at Vivitide (Gardner, MA, USA). Characterization of peptides on LC-MS demonstrated: PSC-PEG-T (*m*/*z*: M+H 1579), DOTA-PEG-T (*m*/*z*: M+H 1580), DOTA-TATE (*m*/*z:* M+3H 478.8). All peptides used in the studies had purity higher than 95%.

### 2.3. Measurement of ^212^Pb Radioactivity

Radioactivity of ^212^Pb was measured on a calibrated Capintec CRC-55R ionization chamber (IC) based dose calibrator (Capintec, Florham Park, NJ, USA). Calibration of the 238 keV gamma-ray peak of ^212^Pb at equilibrium with progeny was carried out on a high-purity germanium (HPGe) detector at the University of Iowa State Hygienic Laboratory (Coralville, IA, USA) using a NIST-traceable ^232^U/^212^Pb source (Cat# 7432, Eckert & Ziegler, Valencia, CA, USA). Once complete, a ^212^PbCl_2_ solution at equilibrium with progeny was measured on the same HPGe in a 5 mL glass ampoule to establish the NIST-traceable radioactivity value. A gravimetric sample of the same ^212^PbCl_2_ solution was then measured on a Capintec CRC-55R to identify instrument calibration settings for ^212^Pb samples in different geometries, including 1.5 mL Eppendorf tubes (Fisher Scientific), 3 mL syringes (Fisher Scientific), 20 mL glass liquid scintillation (LSC) vials (Fisher Scientific), as well as 10 mL, 20 mL and 30 mL glass vials (ALK, Hørsholm, Denmark).

### 2.4. Radiolabeling

Initial evaluation of PSC-PEG-T was conducted by radiolabeling purified-isolated single species of ^212^Pb, ^212^Bi and ^68^Ga. Radiolabeling reactions were conducted using 1 or 5 µM of PSC-PEG-T precursor at different temperatures. Purified ^212^Pb (7.4 MBq) was eluted off a Pb resin chromatography column with 1 M NaOAc buffer (pH 6) into reaction vessels containing 1 or 5 µM PSC-PEG-T precursor and 1 mg mL^−1^ sodium acetate. Final pH was adjusted to 5.4–5.5 by adding pH 4 sodium acetate and reactions were conducted at predetermined temperatures. Radiolabeling of ^212^Bi in PSC-PEG-T was conducted using ^212^Bi purified into single species on RE2 resin (i.e., isolated from ^212^Pb), which comprises organic extractant N,N-diisobutylcarbamoyl-methylphosphine oxide (CMPO) to selectively retain trivalent Bi(III) [33]. Upon purification, generator eluate was loaded on a SPE column filled with 50 mg RE2 resin. The RE2 resin was then rinsed with 1 mL of 2 M HCl containing 1 mg sodium ascorbate to remove Fe(III) by reducing it to Fe(II) [34]. Then, 7.4 MBq of purified ^212^Bi was eluted into the reaction vessel containing 1 or 5 µM PSC-PEG-T in 1 M NaOAc buffer at final pH 5.4. Radiolabeling of ^68^Ga was conducted in 0.5 M sodium acetate buffer using ^68^Ga purified by cation exchange resin as previously described [35]. Thus, 74 MBq ^68^GaCl_3_ from IRE ELIT Galli Eo™ Ge/Ga generator (Fleurus, Belgium) was purified on a Telos SCX column and eluted off the column by 0.5 mL 5.5 M NaCl/ 0.1 M HCl into the reaction vessel containing PSC-PEG-T or DOTA-PEG-T in 2 mL of 0.5 M NaOAc at pH 4, 5 or 6. Further evaluation of PSC-PEG-T was conducted by reacting PSC-PEG-T with mixture of ^212^Pb and ^212^Bi that had reached equilibrium. Then, 7.4 MBq ^212^Pb and ^212^Bi in generator eluate were passed through and preserved on Pb resin and RE2 resin, respectively. Both ^212^Pb and ^212^Bi were simultaneously stripped off the resins by 2 mL 1 M NaOAc buffer (pH 6) into reaction with 5 µM peptide precursor (i.e., PSC-PEG-T, DOTA-PEG-T and DOTA-TATE) as described above. All reactions were conducted at 80 °C for 15 min, followed by analysis of radiochemical yield (RCY) by radio-iTLC method.

### 2.5. Stability of Radiometal Complex and Radiopeptides

Using [^203^Pb]Pb-PSC-PEG-T surrogate, radiochemical stability of [^203^Pb]Pb-PSC-PEG-T in saline was monitored for 72 h. Similarly, single species of [^212^Bi]Bi-PSC-PEG-T in saline were also monitored for 5 h (five half-lives of ^212^Bi). [^203^Pb]Pb-PSC-PEG-T and [^212^Bi]Bi-PSC-PEG-T were radiolabeled and purified on Sep-Pak^®^ C18 SPE columns as previously described [18]. Next, 7.4 MBq mL^−1^ of purified [^203^Pb]Pb-PSC-PEG-T and [^212^Bi]Bi-PSC-PEG-T were stored in saline supplemented with 5% EtOH and 1 mg mL^−1^ sodium ascorbate for 72 h and 5 h, respectively, followed by analysis of radiochemical purity by radio-iTLC method. Then, radiochemical stability of [^212^Pb]Pb-PSC-PEG-T and progeny [^212^Bi]Bi-PSC-PEG-T were determined by radio-iTLC after storage of 37 MBq mL^−1^ radiolabeled [^212^Pb]Pb-PSC-PEG-T in saline for 24 h, and further confirmed by radio-HPLC analysis. In human serum, radiochemical stability of [^212^Pb]Pb-PSC-PEG-T and progeny [^212^Bi]Bi-PSC-PEG-T were determined after incubation of 3.7 MBq [^212^Pb]Pb-PSC-PEG-T in 1 mL human serum at 37 °C for 24 h. In addition, metabolic stability of Pb-PSC-PEG-T radiopeptide in serum was determined by radio-HPLC after incubating [^203^Pb]Pb-PSC-PEG-T surrogate in human serum for 55 h (five half-lives of ^212^Pb). Following the incubation, serum protein was precipitated in ice-cold methanol (1:1.5 *v*/*v*) for 10 min, followed by centrifugation at 10,000× *g* for 10 min. The supernatant was collected and analyzed on radio-HPLC.

### 2.6. Radio-iTLC and Radio-HPLC

In radio-iTLC analysis, 0.1 M NaOAc with 1 mM DTPA was used as mobile phase for ^203^Pb, ^212^Pb, ^212^Bi. Citric acid (0.1 M) was used as mobile phase for ^68^Ga. Upon radio-iTLC analysis, 2 µL aliquot sample was spotted on the radio-iTLC strips (2 × 10 cm) and developed in the mobile phase. Radioactivity of ^203^Pb and ^212^Pb was measured on NaI(Tl) gamma spectroscope using 279 keV and 238 keV gamma peaks, respectively. Radioactivity of ^212^Bi on radio-iTLC was measured on the Ludlum Model 3030 α-particle counter. Radio-HPLC analysis was conducted on Agilent 1260 (Agilent, Santa Clara, CA, USA). Binary mobile phases were applied to run 5–60% phase B (acetonitrile) over phase A (0.1% TFA in water) over 10 min on ZORBAX RR Eclipse XDB-C18 column (4.6 × 150 mm, 5 µm). Radioactivity of ^203^Pb was measured on flow-through 105S-1 single channel radiation detector (Corroll & Ramsey, Fort Collins, CO, USA). To measure ^212^Pb and ^212^Bi activity on radio-HPLC, eluate was collected every 10 s on a HPLC fraction collector. Radioactivity of ^212^Pb and ^212^Bi in each collected eluate was measured on Cobra II automated gamma counter using 238 keV and 583 keV gamma peaks immediately after collection.

### 2.7. In Vivo Biodistribution and SPECT Imaging of [^212^Pb]Pb-PSC-PEG-T

Biodistribution of [^212^Pb]Pb-PSC-PEG-T and progeny ^212^Bi^3+^ was determined in two animal models, including female naïve CD-1Elite mice and athymic mice bearing AR42J tumor xenografts. SSTR2-positve AR42J cells were cultured in 10% Minimum Essential Medium (MEM) supplemented with 10% fetal bovine serum (FBS), 100 units mL^−1^ penicillin, and 100 units mL^−1^ streptomycin 37 °C in a humidified atmosphere (5% CO_2_). All animal studies were performed in accordance with the Guide for the Care and Use of Laboratory Animals, according to protocols approved by the University of Iowa Animal Care and Use Committee (protocol#1122453 approved on 16 February 2022). Xenograft of AR42J tumor was developed by subcutaneous (s.c.) injection of 1 × 10^6^ cells at the left shoulder of female athymic nude mice. [^212^Pb]Pb-PSC-PEG-T was radiolabeled and purified as described above, and kept at room temperature away from light for 3 h before injection, to allow progeny ^212^Bi to approach equilibrium before injection. Upon assay, 74 kBq [^212^Pb]Pb-PSC-PEG-T (peptide mass = 5–10 pmole) were injected via tail vein (n = 2–3). Animals were euthanized at designated time points (1, 5, 24 h for naïve CD-1 mice; 1, 4 h for AR42J-bearing mice). Organs of interest and tumors were collected, rinsed, and weighed. Radioactivity of ^212^Pb and ^212^Bi in each sample was measured on an automated gamma counter by 238 keV and 583 keV gamma peaks, respectively. ^212^Bi was purified from the generator eluate on RE2 resin and collected in pH = 6 sodium acetate as described above. Biodistribution of free ^212^Bi in normal organs was determined at 2 h post injection of 74 kBq ^212^Bi in naïve female CD-1 Elite mice (n = 3). Radioactivity in samples was decay corrected and data were expressed as percent injected dose per gram of tissue (%ID/g). SPECT imaging of [^203^Pb]Pb-PSC-PEG-T and [^212^Pb]Pb-PSC-PEG-T in female athymic nude mice bearing AR42J xenograft (n = 2) was conducted on Bioemtech Gamma-Eye Imaging system (Bioemtech, Athens, Greece) with 30–500 keV dynamic range and 1.9 mm spatial resolution. Whole body 2D-SPECT imaging of [^203^Pb]Pb-PSC-PEG-T and [^212^Pb]Pb-PSC-PEG-T were collected at 3 h and 24 h (n = 2) post injection of 1.8 MBq [^203^Pb]Pb-PSC-PEG-T (52 MBq nmole^−1^) and 3.7 MBq [^212^Pb]Pb-PSC-PEG-T (20 MBq nmole^−1^). SPECT images were generated by acquiring ten 30 s projections. Imaging data were reconstructed and normalized to percent of injected dose (%ID) in tumor and kidneys based on region of interest (ROI) analysis on VISUAL-Eyes software (Bioemtech, Athens, Greece). Animals were euthanized after conclusion of the study at 24 h post injection. Tumor xenografts and organs of interest were collected and weighed. Radioactivity of ^203^Pb or ^212^Pb was determined on automated gamma counter and normalized to %ID/g.

### 2.8. Statistics

Statistical analysis was conducted on GraphPad Prism 8 (GraphPad Software, San Diego, CA, USA). Two-way parametric T-test was used for radiolabeling and stability assays. One-way ANOVA was applied in in vivo experiments.

## 3. Results

### 3.1. Calibration of HPGe and Dose Calibrator for ^212^Pb

Due to the complexity in the decay chain, measurement of ^212^Pb can be complicated by the ingrowth of progeny, among which ^212^Bi and ^208^Tl are the main contributors of x-rays and gamma-rays [36,37]. In this study, the 238 keV gamma peak of ^212^Pb was calibrated on an HPGe detector using a NIST-traceable ^232^U/^212^Pb standard source. The source was measured on an HPGE detector for 10 min to minimize counting uncertainty (σ) to approximately 1%. Dial#760 was identified on a CRC-55R dose calibrator for ^212^Pb samples at equilibrium with daughters in 20 mL LSC vials, 10 mL ALK glass vial and 20 mL ALK glass vial. Dial#790 was identified for 1.5 mL tubes and 3 mL syringe. Dial #672 was identified for 30 mL ALK glass vial. To measure the radioactivity of ^212^Pb prior to equilibrium, time-dependent normalization factors for ^212^Pb samples before equilibrium with progeny (Supplemental Table S1) were established. Using the normalization factors, real-time ^212^Pb radioactivity can be determined using the normalization factor F associated with the time since purification of ^212^Pb. The difference between real activity and readout on the dose calibrator was less than 10% and 2% at 3.5 and 6 h post purification of ^212^Pb, respectively.

### 3.2. Radiolabeling

**Figure 2:**
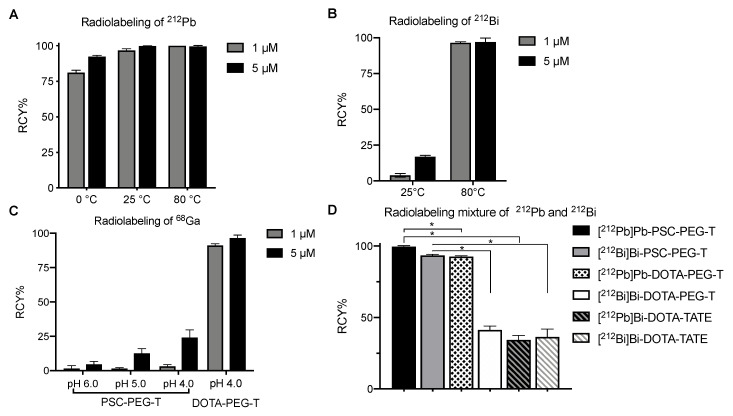
PSC chelator reacts with borderline Pb(II) and Bi(III), but not with hard Ga(III). Radiolabeling reaction yield of single species of (**A**) ^212^Pb, (**B**) ^212^Bi, and (**C**) ^68^Ga^3+^ in PSC-PEG-T was monitored by radio-iTLC method; (**D**) Incorporation of mixture of ^212^Pb and ^212^Bi simultaneously with ^212^Pb/^212^Bi at equilibrium in 5 µM PSC-PEG-T, DOTA-PEG-T and DOTA-TATE. Data presented as mean ± S.D. (n = 2). * *p* < 0.05 by two-tailed *t* test.

Initial evaluation of PSC-PEG-T was conducted by radiolabeling with single-species ^212^Pb, ^212^Bi and ^68^Ga, among which Pb(II) and Bi(III) are borderline Lewis-acids, whereas Ga(III) is considered a hard Lewis-acid metal. We radiolabeled 96.5% of ^212^Pb in PSC-PEG-T at 25 °C within 15 min when the concentration of PSC-PEG-T precursor was 1 µM. Under the same conditions, RCY was increased to 99.8% when 5 µM PSC-PEG-T was used in the reaction (Figure 2A). Under 80 °C, nearly 100% RCY was observed in both 1 and 5 µM PSC-PEG-T within 15 min (Figure 2A). Surprisingly, the high affinity of Pb(II) to PSC-PEG-T chelator-conjugate even enabled radiolabeling at 0 °C. We found 81% and 92% RCY after reaction with 1 and 5 µM PSC-PEG-T for 15 min, indicating the superior affinity between PSC-PEG-T and Pb(II) (Figure 2A). Radiolabeling with ^212^Bi was conducted using purified ^212^Bi in pH 5.4 sodium acetate buffer. Compared with Pb(II), reaction of Bi(III) in PSC-PEG-T required higher temperatures for efficient labeling. At 25 °C, 4% and 17% radiochemical yields were observed with 1 and 5 µM PSC-PEG-T precursor, respectively (Figure 2B). However, when the temperature was elevated to 80 °C, 97% radiolabeling efficiency was found in reactions with both 1 and 5 µM PSC-PEG-T (Figure 2B). Significantly compromised radiochemical yield was observed when PSC-PEG-T was reacted with ^68^Ga. Even under temperatures as high as 95 °C, very minimal ^68^Ga was incorporated in 1 µM PSC-PEG-T under various pH conditions (Figure 2C). In reaction with 5 µM PSC-PEG-T, 25% yield was found under pH 4 (Figure 2C). The radiolabeling efficiency with ^68^Ga was restored in DOTA variant DOTA-PEG-T, suggesting that replacement of the carboxylate to acetamide at 7′ position on cyclen shifts the preference toward bivalent Pb.

Further evaluation of PSC was conducted by radiolabeling a mixture of ^212^Pb and ^212^Bi when the two isotopes were at equilibrium. Using both Pb resin and RE2 resin, 7.4 MBq ^212^Pb and ^212^Bi were purified from generator eluate and collected in 2 mL pH 6 sodium acetate buffer. The breakthrough was less than 1% on each column. After reaction with 5 µM PSC-PEG-T at 80 °C for 15 min, nearly 100% of ^212^Pb and 94% ^212^Bi were incorporated in PSC-PEG-T (Figure 2D). In DOTA-PEG-T, while the incorporation of ^212^Pb was still efficient (RCY = 93%), incorporation of ^212^Bi was significantly compromised (RCY = 41%) (Figure 2D). In DOTA-TATE, introduction of a mixture of ^212^Pb and ^212^Bi simultaneously not only reduced the RCY of [^212^Pb]Pb-DOTA-TATE to 34%, but also decreased RCY of [^212^Bi]Bi-DOTA-TATE to 36% (Figure 2D). The largely compromised radiolabeling efficiency of both ^212^Pb and ^212^Bi in DOTA-conjugated peptides (i.e., DOTA-PEG-T and DOTA-PEG-TATE) is presumably due to the co-existing ^212^Bi and ^212^Pb interfering with each other in the reactions.

### 3.3. Stability of Radiocomplex and Radiopeptides in Saline and Serum

**Figure 3:**
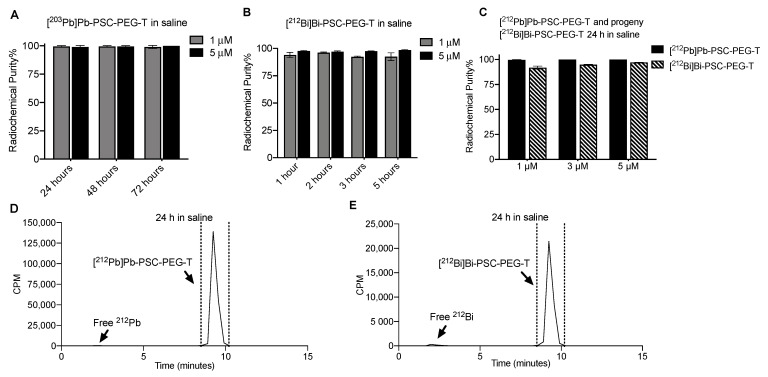
Stability of radiometal and radiopeptide in saline. Radiochemical stability of single species (**A**) [^203^Pb]Pb-PSC-PEG-T and (**B**) [^212^Bi]Bi-PSC-PEG-T with different concentrations of PSC-PEG-T precursor after storage in saline for 24 h (mean ± SD; n = 2); (**C**) Radiochemical stability of ^212^Pb and progeny daughter ^212^Bi in PSC-PEG-T (1, 3, 5 µM) after 24 h in saline; Radio-HPLC analysis of (**D**) [^212^Pb]Pb-PSC-PEG-T and (**E**) progeny [^212^Bi]Bi-PSC-PEG-T after storage in saline for 24 h. In these assays, the radiotracers were stored in saline with 6% EtOH and 1 mg·mL^−1^ sodium ascorbate.

Initial evaluation of the stability of Pb-PSC-PEG-T and Bi-PSC-PEG-T (1 and 5 µM) radiocomplexes was conducted after incubation of single species [^203^Pb]Pb-PSC-PEG-T and [^212^Bi]Bi-PSC-PEG-T in saline. Due to the longer half-life of ^203^Pb, the stability of [^203^Pb]Pb-PSC-PEG-T could be monitored up to 72 h, whereas the stability of [^212^Bi]Bi-PSC-PEG-T was monitored for 5 h, accounting for five half-lives of ^212^Bi. More than 99% of ^203^Pb remained in PSC-PEG-T with less than 1% of free ^203^Pb observed after 72 h storage of [^203^Pb]Pb-PSC-PEG-T in saline regardless of the concentration of PSC-PEG-T precursor (Figure 3A). For [^212^Bi]Bi-PSC-PEG-T, with 1 µM of PSC-PEG-T precursor in incubation, the radiochemical purity was 94.1%, 96.1%, 92.4% and 92.3% after 1, 2, 3 and 5 h incubation. Improved radiochemical purity after 5 h incubation in saline (>98%) was observed when the concentration of PSC-PEG-T precursor was increased to 5 µM (Figure 3B). Radiochemical stability of ^212^Pb and progeny ^212^Bi in PSC-PEG-T was determined after incubating 37 MBq mL^−1^ of [^212^Pb]Pb-PSC-PEG-T in saline at room temperature for 24 h. More than 99.6% of ^212^Pb remained in PSC-PEG-T regardless of PSC-PEG-T concentration. On the other hand, 91.8%, 94.8% and 97.1% of radiochemical purities were found in progeny [^212^Bi]Bi-PSC-PEG-T with 1, 3 and 5 µM of PSC-PEG-T, respectively, after 24 h in saline (Figure 3C), as analyzed by radio-iTLC. The stability of ^212^Pb and ^212^Bi in PSC-PEG-T was also confirmed by the radio-HPLC method (radiochemical purity >95%; Figure 3D,E). The majority (>95%) of ^212^Pb and ^212^Bi remained incorporated as [^212^Pb]Pb-PSC-PEG-T and [^212^Bi]Bi-PSC-PEG-T radiopeptides, suggesting not only stability of both radiometals, but also minimal degradation of the PSC-PEG-T peptide from radiolysis.

**Figure 4:**
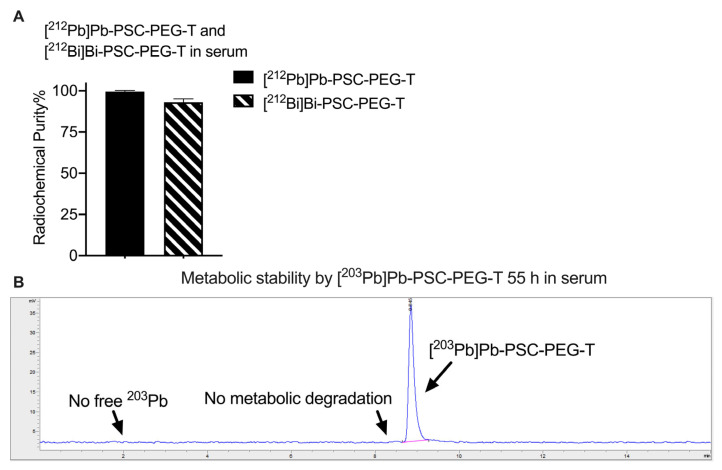
Stability of Pb-PSC-PEG-T in human serum. **(A)** Radio-iTLC analysis of radiochemical purity of [^212^Pb]Pb-PSC-PEG-T and progeny [^212^Bi]Bi-PSC-PEG-T after incubation in serum for 24 h (n = 2); **(B)** Representative radio-HPLC chromatogram of [^203^Pb]Pb-PSC-PEG-T incubation in serum for 55 h.

The stability of [^212^Pb]Pb-PSC-PEG-T and progeny [^212^Bi]Bi-PSC-PEG-T in human serum was determined by radio-iTLC after incubating 3.7 MBq [^212^Pb]Pb-PSC-PEG-T in 1 mL human serum at 37 °C for 24 h. Radiochemical purities of 99.5% and 93.1% were observed for [^212^Pb]Pb-PSC-PEG-T and progeny [^212^Bi]Bi-PSC-PEG-T, respectively (Figure 4A). In addition, using the [^203^Pb]Pb-PSC-PEG-T surrogate with longer decay half-life, the metabolic stability of Pb-PSC-PEG-T radiopeptide was determined by radio-HPLC after incubation of 3.7 MBq [^203^Pb]Pb-PSC-PEG-T in serum for 55 h (five half-lives of ^212^Pb). As shown in Figure 4B, no free ^203^Pb or radiopeptide fragments were observed, suggesting excellent metabolic stability of [^203^Pb]Pb-PSC-PEG-T. Collectively, PSC-PEG-T not only rapidly reacts with ^212^Pb and ^212^Bi, but also preserves the incorporated radiometals in the chelator.

### 3.4. Biodistribution and Micro-SPECT Imaging

**Figure 5:**
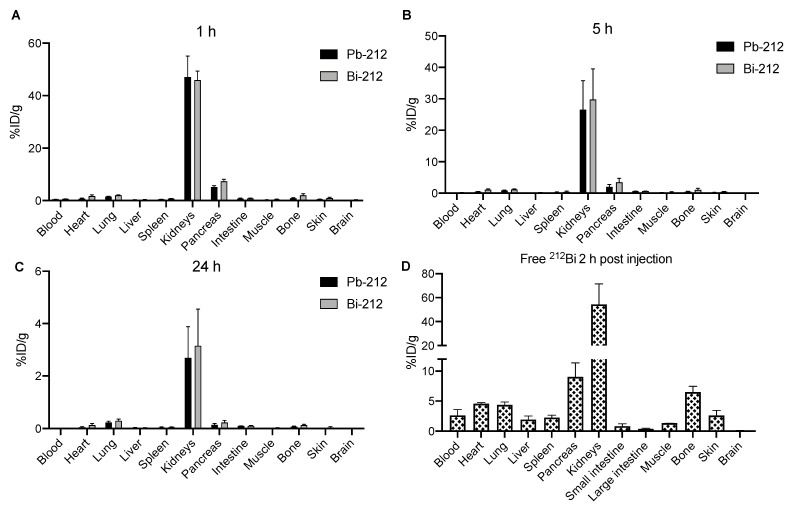
In vivo biodistribution of [^212^Pb]Pb-PSC-PEG-T in naïve tumor free female CD-1 Elite mice at (**A**) 1 h, (**B**) 4 h, and (**C**) 24 h post injection of 74 kBq [^212^Pb]Pb-PSC-PEG-T via tail vein; [^212^Pb]Pb-PSC-PEG-T and [^212^Bi]Bi-PSC-PEG-T were co-injected together when ^212^Bi had reached equilibrium with ^212^Pb. In addition, biodistribution of (**D**) free ^212^Bi was also determined at 2 h post injection of 74 kBq ^212^Bi. Radioactivity of ^212^Pb and ^212^Bi in organs of interest was measured on an automated gamma counter using 238 keV and 583 keV gamma peaks, respectively. Data presented as mean %ID/g ± SD (n = 3).

Biodistribution of [^212^Pb]Pb-PSC-PEG-T was determined in two animal models, including naïve CD1-Elite mice and athymic nude mice bearing AR42J xenografts. In tumor-free CD-1 Elite mice, fast clearance from blood circulation was observed for both [^212^Pb]Pb-PSC-PEG-T and progeny [^212^Bi]Bi-PSC-PEG-T. By 1 h post injection, less than 0.2%ID/g of ^212^Pb and ^212^Bi activities remained in blood (Figure 5A). Both [^212^Pb]Pb-PSC-PEG-T and [^212^Bi]Bi-PSC-PEG-T were cleared through kidneys. Highest accumulation of ^212^Pb activity (47.1 ± 7.9%ID/g) and ^212^Bi activity (45.9 ± 3.7%ID/g) in kidneys was observed at 1 h post injection (Figure 5A), with no significant difference observed between the %ID/g of ^212^Pb and ^212^Bi (*p* > 0.05 by two-way ANOVA). At 5 h post injection, accumulations of ^212^Pb activity and ^212^Bi activity in kidneys decreased to 26.6%ID/g and 29.8%ID/g, respectively (Figure 5B, *p* > 0.05). At 24 h post injection, the residual ^212^Pb activity (2.7 ± 1.2 %ID/g) and ^212^Bi activity (3.1 ± 1.4 %ID/g) in kidneys were low (Figure 5C). In addition, relatively higher accumulation of both ^212^Pb (5.2%ID/g at 1 h; 2.0%ID/g at 5 h) and ^212^Bi (7.3%ID/g at 1 h; 3.5%ID/g at 5 h) in pancreas was found at earlier timepoints, presumably due the SSTR2 expression in pancreas [38]. To determine the biodistribution of free ^212^Bi, 74 kBq ^212^Bi acetate were injected in 100 µL 0.9% sodium chloride in CD-1 Elite mice via tail. Compared with progeny ^212^Bi resulted from the decay of [^212^Pb]Pb-PSC-PEG-T, free ^212^Bi showed much longer biological half-life in vivo, resulting in 2.6%ID/g residue in blood at 2 h post injection (Figure 5D). Free ^212^Bi primarily accumulated in kidneys (54.5 %ID/g; Figure 5D). In addition, significant accumulation of free ^212^Bi was also found in heart (4.6 %ID/g), pancreas (9.0 %ID/g) and bones (6.5 %ID/g) (Figure 5D). These data demonstrate significantly different biodistribution profiles of the progeny ^212^Bi from the decay of [^212^Pb]Pb-PSC-PEG-T and free ^212^Bi^3+^, indicating that progeny ^212^Bi follows the biodistribution of parent [^212^Pb]Pb-PSC-PEG-T radiopeptide with minimal redistribution.

**Figure 6:**
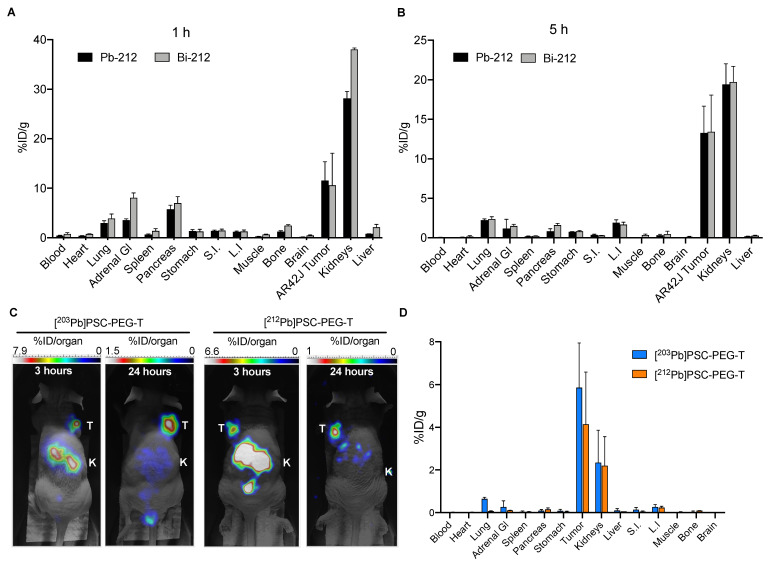
Biodistribution and micro-SPECT imaging in female athymic nude mice bearing AR42J xenograft. Biodistribution of [^212^Pb]Pb-PSC-PEG-T and progeny [^212^Bi]Bi-PSC-PEG-T at (**A**) 1 h and (**B**) 5 h post injection of 74 kBq [^212^Pb]Pb-PSC-PEG-T via tail vein. [^212^Pb]Pb-PSC-PEG-T and [^212^Bi]Bi-PSC-PEG-T were co-injected when ^212^Bi reached equilibrium with ^212^Pb. Data presented as mean %ID/g ± SD (n = 2); SPECT imaging was generated at 3 and 24 h post injection of (**C**) 1.85 MBq [^203^Pb]Pb-PSC-PEG-T and 3.7 MBq [^212^Pb]Pb-PSC-PEG-T (T: tumor; K: kidneys; n = 2); (**D**) Post-imaging biodistribution analysis was conducted to confirm the similar %ID/g of ^203^Pb and ^212^Pb in tumor and organs.

Further analysis of biodistribution of [^212^Pb]Pb-PSC-PEG-T and progeny ^212^Bi was conducted in athymic mice bearing SSTR2-positive AR42J xenografts. Tumor and organs of interest were harvested at 1 h and 4 h post injection. Compared with tumor-free CD-1 Elite mice, accumulations of ^212^Pb and progeny ^212^Bi in kidneys were lower in this model at both 1 h (Figure 6A) and 4 h (Figure 6B), presumably due to “tumor-sink effects”. Similar accumulations of ^212^Pb and ^212^Bi activity were found in the majority of organs and in AR42J tumor xenograft, despite that higher %ID/g of ^212^Bi than ^212^Pb was found in adrenal glands (8.1 vs. 3.6%ID/g), spleen (1.4 vs. 0.6%ID/g), bones (2.5 vs. 1.4%ID/g), kidneys (38.1 vs. 28.1%ID/g) and liver (2.1 vs. 0.8%ID/g) at 1 h post injection (Figure 6A) which might be attributable to different biodistribution between [^212^Pb]Pb-PSC-PEG-T and [^212^Bi]Bi-PSC-PEG-T radiopeptides. However, no difference between the %ID/g of ^212^Pb and ^212^Bi activity was observed at 4 h post injection (Figure 6B).

To confirm the theranostic potential of ^203^Pb and ^212^Pb, whole-body 2D micro-SPECT imaging of [^203^Pb]Pb-PSC-PEG-T and [^212^Pb]Pb-PSC-PEG-T was conducted at 3 and 24 h (n = 2) post injection of 1.85 MBq of [^203^Pb]Pb-PSC-PEG-T or 3.7 MBq of [^212^Pb]Pb-PSC-PEG-T in female athymic nude mice bearing AR42J xenograft (tumor size around 150 mm^3^). Excellent tumor targeting and fast clearance were found for both [^203^Pb]Pb-PSC-PEG-T (tumor 1.8%ID; kidneys: 8.3%ID) and [^212^Pb]Pb-PSC-PEG-T (tumor: 1.2%ID; kidneys: 6.6%ID) at 3 h post injection, resulting in minimal uptake in other normal organs (Figure 6C). At 24 h post injection, prolonged accumulation in tumor and minimal retention in kidneys were observed for both [^203^Pb]Pb-PSC-PEG-T (1.5%ID in tumor; 0.8%ID in kidneys) and [^212^Pb]Pb-PSC-PEG-T (0.9%ID in tumor; 1%ID in kidneys; Figure 6C). Animals were euthanized after conclusion of the assays at 24 h. Radioactivity of ^203^Pb or ^212^Pb in tumors and organs was measured on automated gamma counter and normalized to %ID/g. Despite slight difference in lungs (0.6%ID/g versus 0.3%ID/g), no significant difference between [^203^Pb]Pb-PSC-PEG-T and [^212^Pb]Pb-PSC-PEG-T was found in other analyzed samples including tumors and kidneys (Figure 6D), suggesting the potential of ^203^Pb and ^212^Pb theranostic isotopes for image-guided α-RLT.

## 4. Discussion

In this study, we describe the synthesis and evaluation of a cyclen-based Pb specific chelator (PSC) that contains a mixture of amide and carboxylate donor ligands to improve the in vitro and in vivo stability of ^212^Pb and progeny ^212^Bi in radiopeptides. PSC was conjugated on an octreotide analog via PEG2 linker (PSC-PEG-T) for targeting SSTR2. DOTA-conjugated octreotide analogs including DOTA-PEG-T and DOTA-TATE (i.e., precursor for LUTATHERA^®^) were also synthesized and evaluated as comparison. On the dose calibrator, serial time-dependent normalization factors were determined for ^212^Pb samples prior to equilibrium with daughters. Of note, the first normalization factor was determined at 25 min post purification of ^212^Pb because ^208^Tl (t_1/2_ = 3 min) has moderate retention on the chromatography Pb resin that was used to isolate Pb from other isotopes, resulting in approximately 20% residual ^208^Tl mixed with purified ^212^Pb [18,39]. The residual ^208^Tl resulted in interfered reading on the dose calibrator due to its strong γ-ray emissions (Table 1) from the residual ^208^Tl. Therefore, initial measurement of ^212^Pb activity on the dose calibrator was conducted at 25 min after purification of ^212^Pb to allow complete decay of residual ^208^Tl before measurement. Beyond 6 h post purification of ^212^Pb on Pb resin, all progeny daughters reached near equilibrium with ^212^Pb, resulting in less than 2% difference between A_Real_ activity and A_Read_ under predetermined settings.

Initial radiolabeling of PSC-PEG-T was conducted with purified ^212^Pb, ^212^Bi and ^68^Ga. Pure ^212^Bi was isolated into single species from parent ^212^Pb on RE2 resin. In these reactions, temperature and pH conditions were chosen based on previously reported radiolabeling of ^212^Pb [18,29,40,41,42], and ^213^Bi [43], and ^68^Ga [35]. The high affinity allowed for incorporation of ^212^Pb in 1 µM PSC-PEG-T precursor under temperature as low as 0 °C. Successful radiolabeling of ^212^Bi was also observed in PSC-PEG-T, but only under higher temperature (not room temperature), suggesting relatively lower affinity in PSC compared with ^212^Pb. Poor radiolabeling yield of ^68^Ga was restored in DOTA-PEG-T variant, indicating that the preference toward ^212^Pb and ^212^Bi over ^68^Ga is driven by the diagonal acetyl amide in PSC. Pb cation is a typical borderline Lewis-acid metal [44,45] and Bi cation has been categorized as soft-to-borderline Lewis-acid metal [45,46]. We hypothesize that mixed donor ligands of carboxylate and amide form zero net charge with Pb(II) cation while also maintaining affinity with Bi that allows for >96% radiolabeling efficiency of ^212^Bi at 80 °C within 15 min. Both [Pb]DOTA and [Pb]TCMC chelates are tetragonal antiprism formed by C and N on the cyclen ring [47,48]. PSC shares the same cyclen ring with mixture of amide and carboxylate donor ligands, placing PSC in the middle place on the spectrum between DOTA and TCMC. Therefore, a similar tetragonal antiprism Pb(II) coordination structure is expected in PSC. High radiochemical yield of ^212^Pb and ^212^Bi in PSC-PEG-T was observed not only with pure ^212^Pb and ^212^Bi, but also with mixed ^212^Pb and ^212^Bi that had reached equilibrium. On the other hand, in DOTA-PEG-T and DOTA-TATE, the presence of ^212^Bi resulted in compromised radiolabeling of both ^212^Pb and ^212^Bi. When pure ^212^Pb was introduced into reaction vessels immediately after purification on the Pb resin, minimal progeny ^212^Bi was present in the reaction vessel. By the end of the 15 min reaction, the activity of ^212^Bi (A_Bi-212_) reached 16% of A_Pb-212_ based on progeny ingrowth model [19]. The molar ratio between the number of ^212^Pb atoms (N_Pb-212_) and N_Bi-212_ was 160:1. Upon equilibrium between ^212^Pb and ^212^Bi, the molar ratio between N_Pb-212_ and N_Bi-212_ increased to 10:1. However, in the scale of the reactions conducted in this study, the molar concentrations of peptide precursors were approximately 7400- and 78,000-fold more than ^212^Pb and ^212^Bi, respectively. Therefore, the compromised radiolabeling efficiency was unlikely due to the competition between the two radiometals for precursor, and thus needs to be further elucidated. With these data, we hypothesize that “fresh” ^212^Pb with minimal progeny ^212^Bi is preferred over “aged” ^212^Pb (i.e., with significant buildup of progeny ^212^Bi) if DOTA-conjugated peptides are used.

Stability assays of PSC-PEG-T were conducted in saline and human serum. In initial stability assays, radiochemical stability of radiometal chelates of Pb(II) and Bi(III) in PSC-PEG-T were determined using [^203^Pb]Pb-PSC-PEG-T and [^212^Bi]Bi-PSC-PEG-T as single species, respectively. The longer half-life of ^203^Pb allows for monitoring the stability of [^203^Pb]Pb-PSC-PEG-T in saline up to 72 h. Likewise, metabolic stability of radiometal chelates and radiopeptide were determined after incubating [^203^Pb]Pb-PSC-PEG-T in serum for 55 h. In the stability assays for [^212^Pb]Pb-PSC-PEG-T, the concentration of [^212^Pb]Pb-PSC-PEG-T was 37 MBq mL^−1^ in saline by formulating 370 MBq [^212^Pb]Pb-PSC-PEG-T end product in 10 mL saline with 5% EtOH and 1 mg kg^−1^ sodium ascorbate. This activity concentration was selected to be clinically relevant, based on the injected radioactivity in recently reported clinical trials of ^212^Pb-labeled radiopharmaceuticals, in which 111–150 MBq (i.e., 3–4 mCi) ^212^Pb-labeled end products were administrated per cycle [29,30]. In these assays, both radio-iTLC and radio-HPLC were applied. Radio-iTLC was primarily used to determine the stability of Pb(II) and Bi(III) radiometal-chelates, whereas radio-HPLC allows for identification of potential radiolysis and metabolic degradation of radiopeptide.

In vivo studies in the present report were conducted in two animal models including tumor-free naïve CD-1 Elite mice and athymic nude mice bearing AR42J xenografts. Biodistributions of [^212^Pb]Pb-PSC-PEG-T and free ^212^Bi were first determined in tumor-free mice to avoid “tumor-sinking effects”, where significant amount of radiotracer is absorbed by bulky tumors, resulting in reduced accumulation in normal organs and tissues [49,50]. Indeed, the renal accumulation in athymic nude mice bearing AR42J xenograft (average 785 mm^3^) was lower than tumor-free CD-1 mice. Russ and collaborators have demonstrated that Bi(III) has very long biological half-life in vivo in rats, with multiple clearance compartments [51]. Renal compartments cleared 43% of injected activity, with 13 h biological half-life. On the other hand, non-renal compartments cleared 26% of injected activity, with a 122 h biological half-life [51]. Considering the short radioactive half-life of ^212^Bi (1 h), it is likely that the majority of free ^212^Bi is cleared through renal compartment. Higher ^212^Bi than ^212^Pb in kidneys was observed in athymic nude mice, but not CD-1 Elite mice, at 1 h following injection of [^212^Pb]Pb-PSC-PEG-T. As shown in Figure 3E, the majority of ^212^Bi remained incorporated in [^212^Bi]Bi-PSC-PEG-T radiopeptide; thus, the different %ID/g between ^212^Pb and ^212^Bi activities at 1 h post injection was likely due to the different distribution and clearance rates of [^212^Pb]Pb-PSC-PEG-T and [^212^Bi]Bi-PSC-PEG-T. At 4 h post injection, all ^212^Bi activity in the initial injected dose had decayed and thus the measured ^212^Bi activity was generated from decay of [^212^Pb]Pb-PSC-PEG-T in vivo. No difference between the %ID/g of ^212^Pb and ^212^Bi was observed in tumor and normal organs, suggesting that the ^212^Bi daughter from [^212^Pb]Pb-PSC-PEG-T decay remained well co-localized with parent. In addition to kidneys, positive accumulation was found in pancreas, primarily due to positive expression of SSTR2, which is in line with previously reported SSTR2-targeted analogs [52,53]. Subtle change in the structure of SSTR2-targeted peptide analogs, even change in radiometal chelates, can result in different in vitro binding affinity and in vivo performance as previously demonstrated [53,54]. Detailed evaluation of the improvement from incorporating PSC and PEG linkage in bioactivities will be further elucidated in our ongoing studies.

Following the decay of ^212^Bi, the progeny ^212^Po and ^208^Tl are not expected to stay in the chelator. However, due to the extremely short half-life, toxicity resulting from free ^212^Po occurring in the injected dose is very limited. On the other hand, the impact from free ^208^Tl is also very minimal. The recoiled free ^208^Tl from alpha emission of ^212^Bi results in 0.03% and 0.09% increase in the absorbed dose to the red marrow and the kidneys, respectively [55]. In general, free ^208^Tl results in less than 1.2% absorbed dose in any organ or tissue [55]. In the micro-SPECT imaging study, the Bioemtech Gamma-Eye SPECT Imaging system had energy range of 30–500 keV. Therefore, the camera was calibrated to the major gamma peaks of ^203^Pb (29 keV; 81%) and ^212^Pb (238 keV; 46%), whereas the main gamma emissions from ^208^Tl have relatively high energy (510 keV, 583 keV, 860 keV). Therefore, it is unlikely that the gamma emissions from ^208^Tl have interference in the [^212^Pb]Pb-PSC-PEG-T SPECT imaging.

## 5. Conclusions

In this study, we have synthesized and evaluated a novel Pb specific chelator (PSC) conjugated to an octreotide analog (PSC-PEG-T) for ^212^Pb based α-particle radiotherapy for SSTR2-positive tumors. PSC-PEG-T displays high affinity for the SSTR2 receptor for both ^212^Pb and progeny ^212^Bi. Compared with the DOTA variant, both ^212^Pb and ^212^Bi could be rapidly incorporated in PSC-PEG-T and remain in stable chelation in saline and serum. In addition, nearly identical biodistribution profiles of [^212^Pb]Pb-PSC-PEG-T and progeny [^212^Bi]Bi-PSC-PEG-T in normal organs and tumors were observed in two animal models, suggesting the progeny ^212^Bi remains co-localized with parent [^212^Pb]Pb-PSC-PEG-T in vivo with minimal redistribution.

## Figures and Tables

**Table 1:** Nuclear data of ^203^Pb, ^212^Pb and progeny.

Radionuclide	Half-Life	Decay Mode	Energy (Intensity)
Pb-203	51.9 h	ℇ (100%)	γ: 279 keV (81%)
Pb-212	10.6 h	β^−^ (100%)	β^−^: 40.9 keV (5%); 93.3 keV (81.5%); 171.4 keV (13.7%) γ: 238 keV (46.3%)
Bi-212	60.6 min	β^−^ (64.06%) α (35.94%)	β^−^: 833.9 keV (55.4%) α: 6050.8 keV (25.1%)
Po-212	0.3 µs	α (100%)	α: 8784.9 keV (25.1%)
Tl-208	3 min	β^−^ (100%)	β^−^: 441.5 keV (24.2%); 535.4 keV (22.2%); 649.5 keV (13.7%) γ: 510.8 (22.6%); 583.2 keV (85%); 2614.5 keV (99.8%)

## Data Availability

Data presented in this study are available through communication with the corresponding author.

## Supplementary Materials

The following supporting information can be downloaded at: https://www.mdpi.com/article/10.3390/pharmaceutics15020414/s1, Table S1: Time-dependent normalization factors for the measurement of 212Pb radioactivity.
