# Supplementary material for: Preclinical Evaluation of a Lead Specific Chelator (PSC) Conjugated to Radiopeptides for 203Pb and 212Pb-Based Theranostics
Source: Pharmaceutics. 2023 Jan 26;15(2):414. doi: 10.3390/pharmaceutics15020414 (PMC9966725; doi:10.3390/pharmaceutics15020414)
Supplement: Supplementary file 1 [file pharmaceutics-15-00414-s001.zip › pharmaceutics-2128656-supplementary.pdf]

pharmaceutics

Article

Supplementary Material: Preclinical Evaluation of a Lead Specific Chelator (PSC) Conjugated to Radiopeptides for ^{203}Pb and ^{212}Pb-Based Theranostics

Mengshi Li, Nicholas J. Baumhover, Dijie Liu, Brianna S. Cagle, Frédéric Boschetti, Guillaume Paulin, Dongyoul Lee, Zhiming Dai, Ephraim R. Obot, Brenna M. Marks, Ibrahim Okeil, Edwin A. Sagastume, Moustafa Gabr, F. Christopher Pigge, Frances L. Johnson and Michael K. Schultz

Table S1. Time-dependent normalization factors for the measurement of ^{212}Pb radioactivity.

Time since purification of ^{212}Pb	Normalization Factors*
**25 min**	2.65
**30 min**	2.52
**35 min**	2.29
**45 min**	2.04
**55 min**	1.87
**1 h 15 min**	1.59
**1 h 20 min**	1.51
**1 h 35 min**	1.42
**1 h 47 min**	1.33
**1 h 50 min**	1.3
**2 h**	1.28
**2h 8 min**	1.24
**2 h 20 min**	1.21
**2h 30 min**	1.19
**2 h 45 min**	1.14
**3 h**	1.13
**3 h 10 min**	1.12
**3 h 20**	1.1
**3 h 35 min**	1.09
**3 h 40 min**	1.09
**4 h**	1.07
**4 h 30 min**	1.05
**5 h**	1.04
**5 h 30 min**	1.03
**6 h**	1.02
**^{212}Pb/^{212}Bi equilibrium**	1 (Dial# 690)

***Note:**

$$A_{Real} = A_{Read} \times F$$

where A_{Real} is the real radioactivity, A_{Read} is the measured radioactivity under Dial# 760 for in 20 mL LSC vials, 10 mL ALK glass vial and 20 mL ALK glass vial. #790 for 1.5 mL tube and 3 mL syringe. #672 for 30 mL ALK glass vial.
